# The Serum Profile of Hypercytokinemia Factors Identified in H7N9-Infected Patients can Predict Fatal Outcomes

**DOI:** 10.1038/srep10942

**Published:** 2015-06-01

**Authors:** Jing Guo, Fengming Huang, Jun Liu, Yu Chen, Wei Wang, Bin Cao, Zhen Zou, Song Liu, Jingcao Pan, Changjun Bao, Mei Zeng, Haixia Xiao, Hainv Gao, Shigui Yang, Yan Zhao, Qiang Liu, Huandi Zhou, Jingdong Zhu, Xiaoli Liu, Weifeng Liang, Yida Yang, Shufa Zheng, Jiezuan Yang, Hongyan Diao, Kunkai Su, Li Shao, Hongcui Cao, Ying Wu, Min Zhao, Shuguang Tan, Hui Li, Xiaoqing Xu, Chunmei Wang, Jianmin Zhang, Li Wang, Jianwei Wang, Jun Xu, Dangsheng Li, Nanshan Zhong, Xuetao Cao, George F. Gao, Lanjuan Li, Chengyu Jiang

**Affiliations:** 1 State Key Laboratory for Diagnosis and Treatment of Infectious Diseases, First Affiliated Hospital, College of Medicine, Zhejiang University, Hangzhou 310003, China; 2 Collaborative Innovation Center for Diagnosis and Treatment of Infectious Diseases, Hangzhou,China; 3 State Key Laboratory of Medical Molecular Biology, Institute of Basic Medical Sciences, Chinese Academy of Medical Sciences, Beijing 100005, China; 4 National Institute for Viral Disease Control and Prevention, Chinese Center for Disease Control and Prevention, Beijing 102206, China; 5 Department of Infectious Diseases and Clinical Microbiology, Beijing Chao-Yang Hospital, Beijing Institute of Respiratory Medicine, Capital Medical University, 100020, Beijing, China; 6 Hangzhou Center for Disease Control and Prevention, Hangzhou 310021, China; 7 Jiangsu Provincial Center for Disease Control and Prevention, Nanjing 210009, China; 8 Children’s Hospital, Fudan University, Shanghai 201102, China; 9 Laboratory of Protein Engineering and Vaccine, Tianjin Institute of Industrial Biotechnology, Chinese Academy of Sciences, Tianjin 300308, China; 10 CAS Key Laboratory of Pathogenic Microbiology and Immunology, Institute of Microbiology, Chinese Academy of Sciences, Beijing 100101, China; 11 MOH Key Laboratory of Systems Biology of Pathogens, Institute of Pathogen Biology, Chinese Academy of Medical Sciences and Peking Union Medical College, Beijing 100730, China; 12 Guangzhou Institute of Respiratory Disease, State Key Laboratory of Respiratory Diseases, First Affiliated Hospital, Guangzhou Medical University, Guangzhou 510120, China; 13 Shanghai Institutes for Biological Sciences, Chinese Academy of Sciences, Shanghai 200031, China; 14 Chinese Center for Disease Control and Prevention (China CDC), Beijing 102206, China

## Abstract

The novel avian origin influenza A (H7N9) virus has caused severe diseases in humans in eastern China since the spring of 2013. Fatal outcomes of H7N9 infections are often attributed to the severe pneumonia and acute respiratory distress syndrome (ARDS). There is urgent need to discover biomarkers predicting the progression of disease and fatal outcome of potentially lethal flu infections, based on sound statistical analysis. We discovered that 34 of the 48 cytokines and chemokines examined in this study were significantly elevated in the plasma samples from patients infected with H7N9. We report for the first time that the levels of MIF, SCF, MCP-1, HGF, and SCGF-β are highly positively linked to disease severity and the profile of mediators MIF, SCF, MCP-1, HGF, SCGF-β, IP-10, IL-18, and IFN-γ is an independent outcome predictor.

In 2013, 144 people in eastern China had been confirmed infected with influenza A H7N9, a novel avian-origin influenza A reassortant subtype virus, with 48 people reported death^1^,^2^,^3^,^4^,^5^. Hundreds of people infected with influenza A(H7N9) virus have been reported this year. The clinical characteristics of these patients often included fever and cough, pneumonia, and acute respiratory distress syndrome (ARDS)^2^,^6^,^7^.Hypercytokinemia has been reported in the peripheral bloods of both H7N9-infected patients and H5N1-infected patients^7^,^8^,^9^,^10^. Our previous studies have reported that Angiotensin II level was markedly elevated in the plasma from patients infected with H5N1 and H7N9 viruses^11^,^12^. Moreover, the elevated level of Angiotensin II can be a biomarker for the outcome of H7N9 infected patients^11^. Furthermore, we would like to investigate whether elevated cytokines and chemokines in the plasmas of avian influenza infected patients can be better or alternative biomarkers for H7N9 infected patients. Although IFITM3 dysfunctions and elevated IL-6, IL-8, and MIP-1β levels have been reported in avian influenza infected patients, these molecules have been reported in limited number of cases available from the same hospital and have not been studied with comprehensive statistical analyses^9^,^13^,^14^.

We recruited 47 patients infected with H7N9 in Zhejiang and Jiangsu province. 21 patients infected with swine-origin influenza A (H1N1) virus in Beijing during the same period were recruited as control. Most H7N9 infected patients received oseltamivir or peramivir, antibiotic, glucocorticoid or/and immune globulin therapy. In order to eliminate these interference as far as possible, we collected their plasma before or at the beginning of any therapy. We showed that a plasma profile of hypercytokinemia dynamics signature proteins including MIF, SCF, HGF, MCP-1, SCGF-β, IP-10, IL-18, and IFN-γ is dynamic biomarker to predict fatal outcomes. Among them MIF, SCF, MCP-1, and HGF can predict the outcome of H7N9-infected patients better than Angiotensin II.

## Result

### Hypercytokinemia in H7N9-infected patients

We have performed multiplex analyses of 48 cytokine and chemokine mediators using these plasma samples while only 17 of these 48 mediators have been previously examined and reported inpatients infected with avian influenza virus^7^,^8^,^9^.

**Figure 1:**
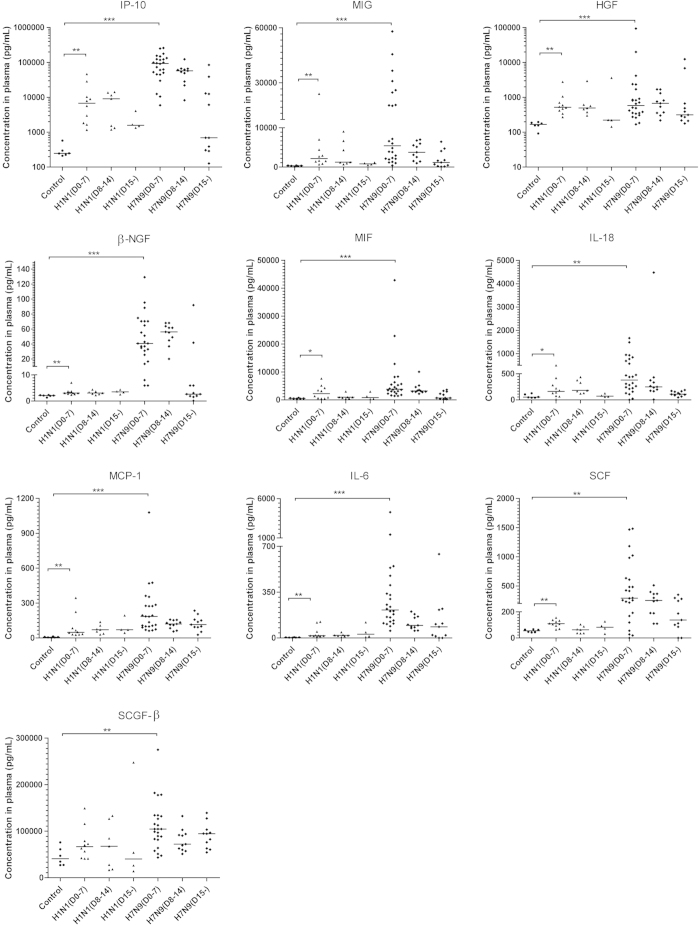
Hypercytokinemia in Avian Influenza A (H7N9) Virus-Infected Patients. A total of 46 patients infected with the avian influenza A (H7N9) virus, 21 patients infected with the swine-origin influenza A (H1N1) virus and 6 healthy controls were recruited for this study. Their plasma levels of chemokines and cytokines were measured. Of the chemokines and cytokines examined, 34 were significantly elevated in the plasma of patients infected with the avian influenza A (H7N9) virus at 0-7 days (n = 24), 8-14 days (n = 11) and 15 days (n = 11) after the onset of the disease. Detailed information is shown in supplementary table 1. *P < 0.05, **P < 0.01, ***P < 0.001.

In general, hypercytokinemia was observed in the plasma from patients infected with avian influenza A (H7N9) virus, but not in the plasma from patients infected with swine-origin influenza A (H1N1) virus (S-OIV)who are the same cohort of our previous study (Fig. 1, Supplemental figure S1, Table S1)^11^. Overall, 34 of 48 measured cytokines and chemokines in the plasma harvested within the first 7 days of the disease onset of H7N9-infected patients were significantly higher than in samples from non-infected control individuals (Fig. 1, Supplemental figure S1). Notably, the elevation of chemokine IP-10, a critical player in the induction of lung injury^15^,^16^,^17^, was the most robust among all of the cytokines and chemokines measured (Fig. 1). Additionally, increased levels of IL-2 and other mediators that participate in the development of T-helper 1, T-helper 2, and T-helper 17 cells were also observed (Fig. 1, Supplemental figure S1)^18^. Similar cytokine/chemokine profiles were noted in patients whose plasmas were collected during the second week between 8 and 14 days after disease onset (Supplemental Table S1). These data confirmed the presence of hypercytokinemia, also known as a “cytokine storm”, in patients infected with the H7N9 virus and discovered 18 novel hypercytokinemia factors that were elevated in these patients, including MIF, SCF, HGF, SCGF-β, IL-18, β-NGF, SDF-1α, CTACK, IL-1ra, IL-9, G-CSF, IL-7, IL-5, GM-CSF, VEGF, IL-13, basic-FGF, and PDGF-bb elevated in avian influenza infected patients plasmas.

The cytokine and chemokine levels in patients’plasma obtained 15 days after disease onset were generally decreased compared with the levels in plasma obtained during the acute phase of the disease. These results suggest that the plasma levels of cytokines and chemokines may be related to the severity of disease (Fig. 1, Supplemental figure S1).

### Some cytokine and chemokine plasma levels are correlated with the viral load of H7N9

From the plasma samples obtained from these 35 patients, we analyzed the potential correlations between the plasma levels of 34 elevated cytokines and chemokines and the patients viral load, APACHE II score^19^, and mortality respectively. We determined the viral loads using the cycle threshold (Ct) values of the H7N9-specific hemagglutinin (HA) gene measured in qRT-PCR as previously described^7^. The plasma levels of IP-10, MIG, MIF, HGF, and IL-18 were negatively correlated with the Ct values of *H7* gene (and thus positively correlated with the viral load) during both week 1 and week 2 after disease onset (Table 1). The plasma levels of SCF, β-nerve growth factor (β-NGF), and SCGF-β were negatively correlated with the Ct values of *H7* gene only during the second week after disease onset (Table 1). These results suggest the existence of hypercytokinemia dynamics that are induced by the H7N9 virus.

### Some cytokine and chemokine levels are related to the severity of disease

The APACHE II scores were used to evaluate the severity of disease on the first day that patients were admitted to the intensive care unit (ICU)^20^. The plasma levels of SCF, HGF, MIF, IL-18, IP-10, and MIG during both week 1 and week 2 after disease onset showed a high and significant positive linear correlation with the APACHE II scores (Table 2, Supplemental figure S2, S3). The levels of HGF, IL-18, SCF, MIG, and IP-10 in plasma samples obtained during the first week of disease onset were especially highly associated with disease severity, indicating these molecules could serve as biomarkers at the early stages of infections (Table 2, Supplemental figure S2). These results suggest that the hypercytokinemia dynamics are correlated with disease severity and that HGF, IL-18, SCF, MIG, and IP-10 may be associated with the severity of disease in the early disease stages.

### Some cytokine and chemokine plasma levels can predict the fatal outcomes

**Figure 2:**
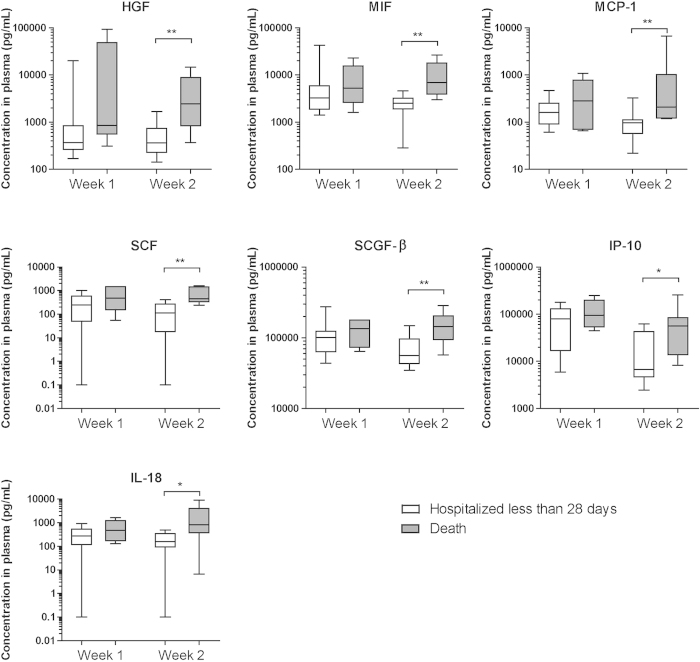
Fatal Outcomes were Associated with Plasma Levels of Chemokines and Cytokines and with Clinical Characteristics in H7N9-Infected Patients. The chemokine and cytokine (HGF, MIF, MCP-1, SCF, SCGF-β, IP-10, IL-18) concentrations in the plasma harvested during the first and second weeks after disease onset compared between different outcome groups are shown. The groups include patients who left the hospital within 28 days (n = 12) and patients who died (n = 5). The plasma samples were collected during the first week of disease onset from patients who left the hospital within 28 days (n = 16) and from patients who died (n = 8) and whose plasma samples were harvested in the second week of disease onset. *P < 0.05, **P < 0.01.

We further analyzed whether these plasma cytokine and chemokine levels were linked to fatal outcomes. We noted that the plasma levels of HGF, MIF, MCP-1, SCF, SCGF-β, IP-10, and IL-18 during the second week of disease were found to be significantly higher in patients who died than in patients who left the hospital within 28 days (Fig. 2, Supplemental Table S2).

**Figure 3:**
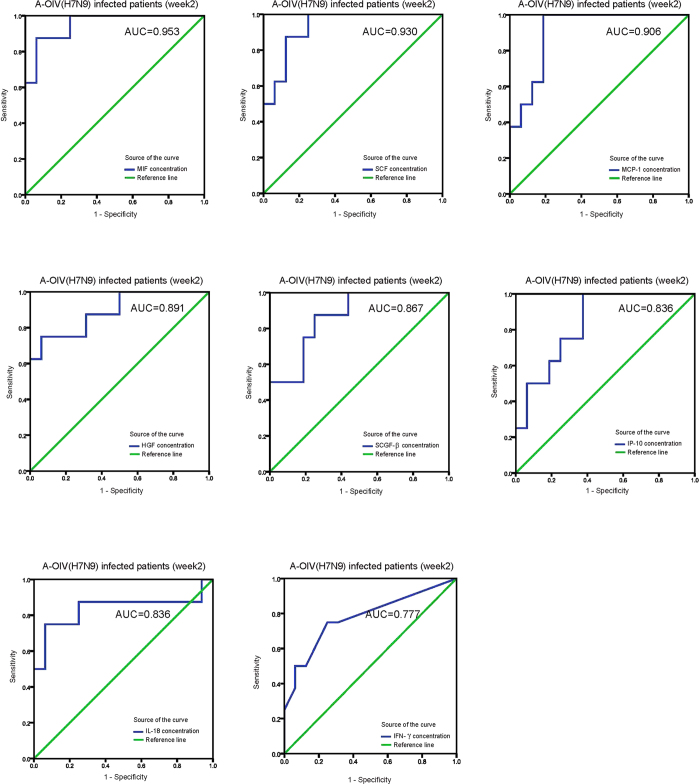
ROC curve of the Plasma Levels of Chemokines and Cytokines and Clinical Characteristics during Week 2 of Disease Onset. The ROC curve of the plasma chemokine and cytokine levels (MIF, SCF, MCP-1, HGF, SCGF-β, IP-10, IL-18, IFN-γ) during week 2 of disease onset. Detailed information on the AUC is shown in supplementary tables 3.

To statistically link each independent inflammatory mediator to the prediction of the fatal outcomes, we calculated the area under the curve (AUC) of the receiver operating characteristics (ROC) curve for all of the elevated cytokines and chemokines induced by H7N9 infection (Supplemental Table S3). The AUC values of the ROC curves of MIF, SCF, MCP-1, HGF, SCGF-β, IP-10, IL-18, and IFN-γ had significant P values (Fig. 3, Supplemental Table S3). Therefore, the levels of these identified proteins in plasma samples harvested from patients infected with H7N9 during the second week of disease onset could predict the fatal outcomes. The P values of the other reported significantly elevated cytokines and chemokines in plasma samples of H7N9-infected patients in this study including β-NGF, IL-6, MIG, IL-8, MIP-1β, SDF-1α, CTACK, MIP-1α, IL-1ra, IL-9, IL-10, G-CSF, IL-7, IL-5, GM-CSF, IL-1β, IL-4, VEGF, TNF-α, IL-13, IL-2, basic-FGF, IL-12p70, PDGF-bb, IL-17A, and RANTES are not significant to predict fatal outcomes (Supplemental table S3). These results suggest that the levels of the hypercytokinemia factors MIF, SCF, MCP-1, HGF, SCGF-β, IP-10, IL-18, and IFN-γ can be biomarkers that predict fatal outcomes for H7N9-infected patients.

We also analyzed the logistic regression of outcome predictor independency with the cytokines and chemokines harvested in the patients’ plasmas during the second week of disease onset. We used genomic mean of fold changes of hypercytokinemia factors levels including MIF, SCF, MCP-1, HGF, SCGF-β, IP-10, IL-18, and IFN-γ in the plasma samples of H7N9-infected patients because these cytokines and chemokines have high associations among themselves (Table 3, Supplemental Figure S4). The profile of these 8 cytokines and chemokines was a statistically independent outcome predictor (Table 3).

## Discussion

It is interesting to note that the inflammatory mediators MIF, SCF, MCP-1, HGF, SCGF-β, IL-18, IP-10, and IFN-γ play distinctly different roles in immune responses^21^,^22^,^23^,^24^,^25^. MIF, MCP-1, and IP-10 are chemokines that can induce directed chemotaxis and regulate functions of monocyte/macrophage, T cells, natural killer cells, and dendritic cells^23^; IL-18 is also called IFN-γ inducing factor, and the important function of IFN-γ is known to inhibit viral replication directly^22^; SCF and SCGF-β play important roles in the survival and self-renewal of hematopoietic stem cells (HSC)^24^, and HGF can stimulate the regeneration of epithelial cells and endothelial cells in addition to acting on hematopoietic progenitor cells^21^. These mechanisms may provide a hypothesis for the molecular bases that are responsible for fatal outcomes of lethal virus induced ARDS.

The biomarkers require an independent cohort for confirmation. SCF is already used in major hospitals in China as a diagnostic biomarker to monitor disease progress of non-small-cell lung cancer^26^. Because of the current dramatic increase in new cases of H7N9-infected patients in China, it may be pragmatic to examine the SCF levels in the plasma of patients infected with H7N9 to monitor the severity of disease and to predict the fatal outcomes.

The high elevation of IP-10 has been reported in the plasma of patients with SARS-CoV, avian influenza H5N1, and swine-origin influenza virus infections^15^,^16^,^17^. We compared some other cytokines and chemokines mediators in the plasma of patients with avian influenza H7N9, H5N1, and swine-origin influenza virus infections as well as SARS-CoV infections reported previously. The elevation levels of these mediators in H7N9 patients are generally near to that of H5N1 patients and more significant than that of SARS-CoV and swine flu patients. Nevertheless, the comparison may be disturbed by the sensitivity of different assay kits(Supplemental Table 4)^10^,^15^,^16^,^27^,^28^. Furthermore, the lack report of MIF, SCF, HGF, MCP-1, and SCGF-β elevations in lethal virus infections may be due to the lack of laboratory examinations. Further studies are necessary to demonstrate whether the findings in this study could extend to more lethal virus infections.

## Methods

### Clinical specimens

We recruited 46 patients infected with H7N9, 21 patients infected with H1N1 and 6 healthy volunteers. The flowchart of our recruit and their basic clinical characteristics are described previously^11^. This study was approved by the institutional review board of the First Affiliated Hospital of Zhejiang University, Beijing Chao-yang Hospital and China CDC. The Declaration of Helsinki was strictly followed. The methods were carried out in accordance with the approved guidelines. Human pharyngeal swabs (or sputum specimens) and peripheral venous blood from all patients were collected in specimen tubes with Hank’s buffer on the first day of admission and throughout the course of the illness. The laboratory confirmation of H7N9 virus and H1N1 virus infection were performed using the protocols described previously^2^,^6^,^7^. We obtained written informed consent from all of the participants or their guardians.

### RNA extraction and real-time RT-PCR

We extracted the RNA from the samples using the RNeasy Mini Kit (QIAGEN, Germany) within 24 h after sampling. We performed the real-time RT-PCR experiments using the influenza A (H7N9) virus subtype-specific *H7* gene primers provided by the China CDC in an ABI7500 (ABI, USA)^2^. The data were analyzed if they passed the quality controls. The specimens with Ct values ≤ 38.0 were considered positive. The specimens with Ct >38 were repeated. The specimens with repeated results that also gave Ct values >38 were considered positive, and the specimens with undetectable Ct values after the repeat were considered negative.

### Cytokine and chemokine measurements

We collected the plasma of patients with the laboratory confirmation of H7N9 virus or H1N1 virus infection and measured the plasma levels of 48 cytokines and chemokines with the Bio-Plex Pro^TM^ Human Cytokine Array 27-Plex Group I and 21-Plex Group II Kits on a Luminex200^TM^ (Luminex^®^Multiplexing Instrument, Merck Millipore) in a BSL-2 laboratory following the manufacturers’ instructions. Then, we analyzed the raw data using xPONENT 3.1 software (Merck Millipore).

### Statistical analysis

We used Mann-Whitney U-tests to determine whether the differences in the cytokine and chemokine levels between groups were statistically significant. We assumed a value of 0.1 pg/mL for statistical purposes in cases in which the concentration was undetectable. We used Spearman’s rank correlation coefficient analysis to analyze the linear correlation. We used the Benjamini & Hochberg method to control the false discovery rate (FDR) for multiple testing corrections. We calculated the ROC curves for predictive analysis. We used logistic regression to analyze the independence of outcome predictors. The Mann-Whitney U-tests, correlation statistical analysis, ROC curve analysis and logistic regression were performed using SPSS16.0. The multiple testing correction FDR values were calculated using Software R. A *P* value of less than 0.05 derived from a two-tailed test of all analyses was considered statistically significant.

## Additional Information

**How to cite this article**: Guo, J. *et al.* The Serum Profile of Hypercytokinemia Factors Identified in H7N9-Infected Patients can Predict Fatal Outcomes. *Sci. Rep.* **5**, 10942; doi: 10.1038/srep10942 (2015).

## Supplementary Material

Supplementary Information

## Figures and Tables

**Table 1:** Ct Values of the Highly Related Cytokine/Chemokines in Patients Infected with H7N9.

	**Week1 (N=21)**			**Week2 (N=30)**		
**Cyto/Chemokines**	**Spearman**	**P Value**	**FDR**	**Spearman**	**P Value**	**FDR**
IP-10	−0.629	0.002	0.020	−0.833	<0.001	<0.001
MIG	−0.507	0.019	0.043	−0.697	<0.001	<0.001
SCF	—	—	—	−0.597	<0.001	0.003
β-NGF	—	—	—	−0.584	0.001	0.002
MIF	−0.486	0.026	0.038	−0.530	0.003	0.006
SCGF-β	—	—	—	−0.501	0.005	0.010
HGF	−0.569	0.007	0.032	−0.492	0.006	0.011
IL-18	−0.475	0.030	0.038	−0.490	0.006	0.010
MCP-1	—	—	—	−0.454	0.012	0.017
IL-6	—	—	—	−0.399	0.029	0.033

**Table 2:** APACHE II Score of Highly Related Cytokine/Chemokines in Patients Infected with H7N9.

	**Week1(N=21)**			**Week (N=2)**		
**Cyto/Chemokines**	**Spearman**	**P Value**	**FDR**	**Spearman**	**P Value**	**FDR**
SCF	0.656	0.001	0.003	0.800	<0.001	<0.001
HGF	0.747	0.000	0.000	0.796	<0.001	<0.001
SCGF-β	—	—	—	0.713	<0.001	<0.001
MIF	0.446	0.043	0.047	0.648	<0.001	<0.001
IL-18	0.704	0.000	0.001	0.607	<0.001	0.001
IP-10	0.600	0.004	0.007	0.575	0.001	0.001
MIG	0.643	0.002	0.004	0.557	0.001	0.002
IL-6	—	—	—	0.483	0.005	0.009
MCP-1	0.439	0.046	0.046	0.463	0.008	0.011
β-NGF	0.499	0.021	0.032	0.430	0.014	0.019

**Table 3:** Logistic Regression Analysis of Outcome Predictor Independence in Patients Infected with H7N9 during Week 2 of Disease Onset.

	**B**	**S.E.**	**Wald**	**Sig.**	**Exp(B)**	**95.0% C.I. for EXP(B)**	
						**Lower**	**Upper**
Age	0.017	0.089	0.036	0.849	1.017	0.854	1.212
Sex	1.629	1.975	0.680	0.410	5.098	0.106	244.684
Coexisting conditions	19.142	15281.291	0.000	0.999	2.05E10	0.000	—
Cytokine/Chemokines	0.771	0.391	3.890	0.049	2.161	1.005	4.649
Constant	−27.876	15281.291	0.000	0.999	0.000	—	—
