# Corrigendum: The Serum Profile of Hypercytokinemia Factors Identified in H7N9-Infected Patients can Predict Fatal Outcomes

**DOI:** 10.1038/srep21230

**Published:** 2016-02-23

**Authors:** Jing Guo, Fengming Huang, Jun Liu, Yu Chen, Wei Wang, Bin Cao, Zhen Zou, Song Liu, Jingcao Pan, Changjun Bao, Mei Zeng, Haixia Xiao, Hainv Gao, Shigui Yang, Yan Zhao, Qiang Liu, Huandi Zhou, Jingdong Zhu, Xiaoli Liu, Weifeng Liang, Yida Yang, Shufa Zheng, Jiezuan Yang, Hongyan Diao, Kunkai Su, Li Shao, Hongcui Cao, Ying Wu, Min Zhao, Shuguang Tan, Hui Li, Xiaoqing Xu, Chunmei Wang, Jianmin Zhang, Li Wang, Jianwei Wang, Jun Xu, Dangsheng Li, Nanshan Zhong, Xuetao Cao, George F. Gao, Lanjuan Li, Chengyu Jiang

Scientific Reports 5: Article number: 1094210.1038/srep10942; published online: 06012015; updated: 02232016

This Article contains typographical errors in Table 2 where ‘Week 2 (N = 32)’ was incorrectly given as ‘Week (N = 2)’.

