# Supplementary material for: The Serum Profile of Hypercytokinemia Factors Identified in H7N9-Infected Patients can Predict Fatal Outcomes
Source: Sci Rep. 2015 Jun 1;5:10942. doi: 10.1038/srep10942 (PMC4450576; doi:10.1038/srep10942)
Supplement: Supplementary Information [file srep10942-s1.pdf]

Supplementary information

The Serum Profile of Hypercytokinemia Factors Identified in H7N9-Infected Patients can Predict Fatal Outcomes

Jing Guo[1,2]*, Fengming Huang[3]*, Jun Liu[4,5]*,Yu Chen[1,2]*, Wei Wang[3]*, Bin Cao[6]*, Zhen Zou[3], Song Liu[3], Jingcao Pan[7], Changjun Bao[8], Mei Zeng[9], Haixia Xiao[10], Hainv Gao[1,2], Shigui Yang[1,2], Yan Zhao[3], Qiang Liu[3], Huandi Zhou[3], Jingdong Zhu[3], Xiaoli Liu[1,2], Weifeng Liang[1,2], Yida Yang[1,2], Shufa Zheng[1,2], Jiezuan Yang[1,2], Hongyan Diao[1,2], Kunkai Su[1,2], Li Shao[1,2], Hongcui Cao[1,2], Ying Wu[5], Min Zhao[5], Shuguang Tan[5], Hui Li[6], Xiaoqing Xu[3], Chunmei Wang[3], Jianmin Zhang[3], Li Wang[3], Jianwei Wang[11], Jun Xu[12], Dangsheng Li[13], Nanshan Zhong[12], Xuetao Cao[3], George F Gao[2,4,5,14], Lanjuan Li[1,2], Chengyu Jiang[3]

[1]State Key Laboratory for Diagnosis and Treatment of Infectious Diseases, First Affiliated Hospital, College of Medicine, Zhejiang University, Hangzhou 310003, China
[2]Collaborative Innovation Center for Diagnosis and Treatment of Infectious Diseases, Hangzhou,China.
[3]State Key Laboratory of Medical Molecular Biology, Institute of Basic Medical Sciences, Chinese Academy of Medical Sciences, Beijing 100005, China.
[4]National Institute for Viral Disease Control and Prevention, Chinese Center for Disease Control and Prevention, Beijing 102206, China
[5]CAS Key Laboratory of Pathogenic Microbiology and Immunology, Institute of Microbiology, Chinese Academy of Sciences, Beijing 100101, China
[6] Department of Infectious Diseases and Clinical Microbiology, Beijing Chao-Yang Hospital, Beijing Institute of Respiratory Medicine, Capital Medical University, 100020, Beijing, China.
[7]Hangzhou Center for Disease Control and Prevention, Hangzhou 310021, China.
[8]Jiangsu Provincial Center for Disease Control and Prevention, Nanjing 210009, China.
[9]Children's Hospital, Fudan University, Shanghai 201102, China.
[10]Laboratory of Protein Engineering and Vaccine, Tianjin Institute of Industrial Biotechnology, Chinese Academy of Sciences, Tianjin 300308, China.
[11]MOH Key Laboratory of Systems Biology of Pathogens, Institute of Pathogen Biology, Chinese Academy of Medical Sciences and Peking Union Medical College, Beijing 100730, China.
[12]Guangzhou Institute of Respiratory Disease, State Key Laboratory of Respiratory Diseases, First Affiliated Hospital, Guangzhou Medical University, Guangzhou 510120, China.
[13]Shanghai Institutes for Biological Sciences, Chinese Academy of Sciences, Shanghai 200031, China.
[14]Chinese Center for Disease Control and Prevention (China CDC), Beijing 102206, China.

* These authors contributed equally to this work.

Correspondence should be addressed to C.Y.J (jiang@pumc.edu.cn), L.J.L (ljli@zju.edu.cn) or G.F.G (gaofu@chinacdc.cn).

Supplementary Figure1

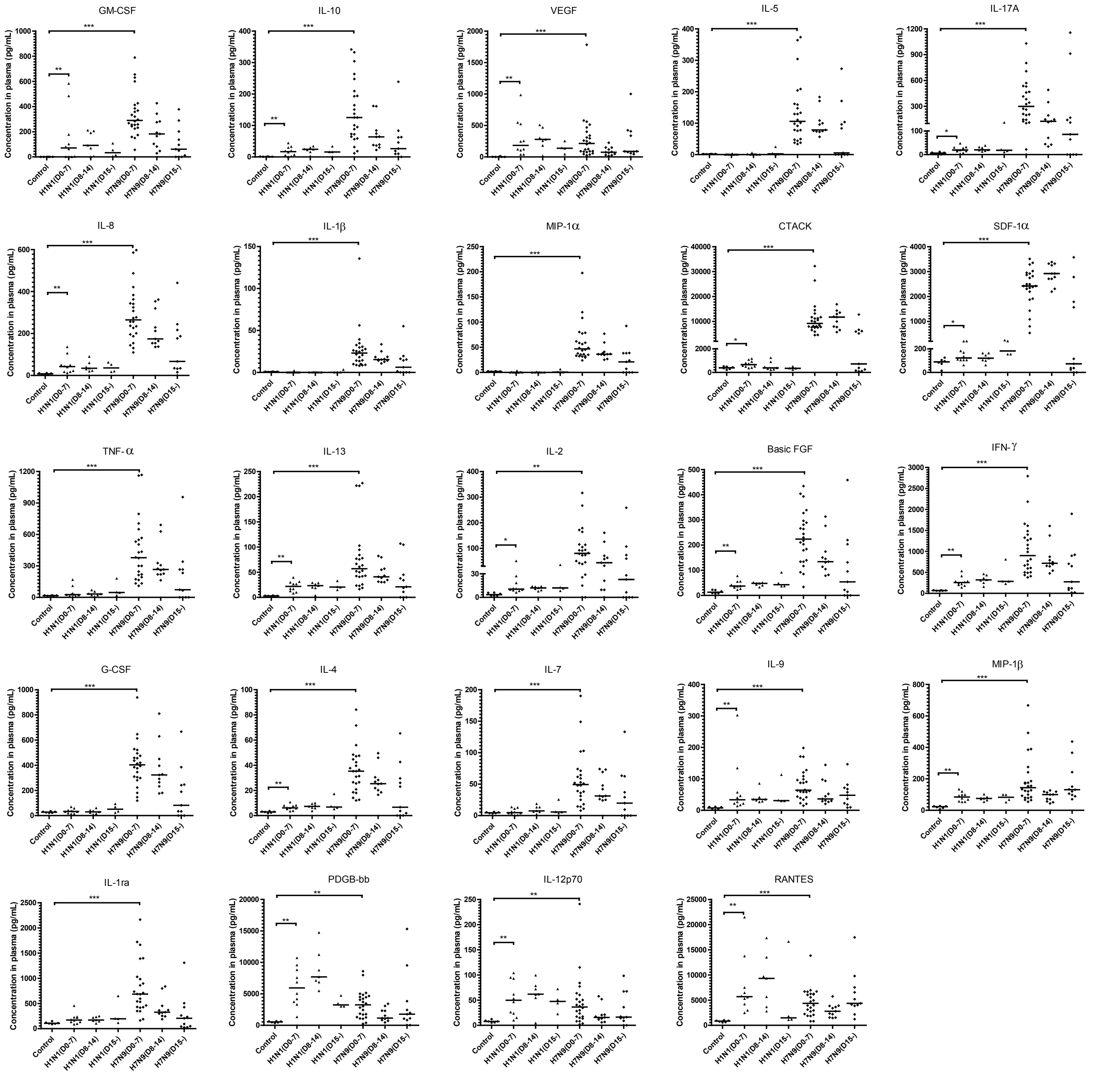

Supplementary Table 1. Cytokine/chemokine Comparison Among 6 healthy controls, 21 H1N1-infected patients and 46 H7N9-infected patients.

	Control（n=6）		H1N1（n=21）												H7N9（n=46）												P^m	P^n	P^o
			Day0-7 (n=10)			Day8-14 (n=7)			Day15- (n=4)			P^d	P^e	P^f	Day0-7 (n=24)			Day8-14 (n=11)			Day15- (n=11)			P^j	P^k	P^l			
	Mean	SD	Mean	SD	P^a	Mean	SD	P^b	Mean	SD	P^c				Mean	SD	P^g	Mean	SD	P^h	Mean	SD	P^i						
GM-CSF	0.08	0.04	149.16	200.64	0.005	110.58	85.43	0.005	44.86	41.04	0.006	0.625	0.887	0.345	339.06	172.28	<0.001	193.73	121.14	<0.001	111.48	122.53	0.007	0.026	<0.001	0.122	0.006	0.221	0.694
IP-10	300.24	125.48	11522.70	13861.96	0.001	7487.00	5533.30	0.003	2144.25	1125.56	0.011	0.922	0.066	0.450	96542.29	66191.64	<0.001	54232.48	28556.77	<0.001	14333.58	24884.23	0.021	0.088	<0.001	0.003	<0.001	0.002	0.794
IL-10	0.79	0.36	17.40	13.77	0.009	21.88	9.48	0.003	16.36	11.73	0.133	0.380	0.990	0.340	144.11	95.86	<0.001	70.49	46.73	<0.001	50.79	65.28	0.035	0.026	0.002	0.094	<0.001	0.002	0.433
VEGF	2.44	4.43	297.86	286.67	0.001	286.67	155.00	0.002	142.91	73.83	0.008	0.626	0.777	0.089	292.95	354.14	<0.001	90.04	61.86	<0.001	237.15	282.28	0.003	0.014	0.356	0.250	0.850	0.016	0.990
IL-6	4.10	1.12	40.32	41.57	0.001	20.73	10.86	0.003	45.91	45.00	0.011	0.660	0.671	0.990	458.11	842.75	<0.001	114.10	49.48	<0.001	130.63	178.33	0.035	0.002	0.006	0.533	<0.001	<0.001	0.514
IL-5	1.71	0.47	0.56	0.83	0.010	1.26	1.79	0.111	7.44	10.04	0.831	0.585	0.082	0.322	133.14	95.03	<0.001	99.01	39.31	<0.001	66.61	86.23	0.760	0.434	0.030	0.209	<0.001	<0.001	0.637
IL-17A	5.53	4.32	21.46	12.37	0.013	23.78	8.51	0.003	39.47	41.46	0.019	0.591	0.777	0.705	348.40	250.25	<0.001	159.73	134.74	<0.001	239.82	382.19	0.263	0.028	0.024	0.374	<0.001	<0.001	0.598
MIG	315.66	109.49	4721.20	6676.86	0.001	3448.07	3067.92	0.003	844.69	246.95	0.011	0.696	0.034	0.089	13436.62	15595.19	<0.001	3981.05	2103.34	<0.001	1959.72	2056.38	0.088	0.241	0.003	0.033	0.076	0.342	0.602
IL-8	7.02	3.79	49.41	38.76	0.001	42.41	23.01	0.003	37.72	19.63	0.011	0.922	0.990	0.705	293.28	128.54	<0.001	217.84	82.97	<0.001	131.89	129.64	0.007	0.095	0.002	0.139	<0.001	<0.001	0.296
HGF	160.94	34.04	770.27	704.51	0.001	823.29	874.19	0.003	1051.16	1480.78	0.134	0.845	0.157	0.186	5547.17	18788.88	<0.001	793.22	514.05	<0.001	2028.33	3785.47	0.002	0.915	0.166	0.158	0.880	0.751	0.361
MCP-1	6.95	3.49	93.83	100.72	0.001	73.70	35.45	0.003	95.08	58.16	0.011	0.495	0.396	0.776	234.30	213.91	<0.001	116.23	33.97	<0.001	124.53	58.85	<0.001	0.110	0.136	0.922	0.002	0.042	0.296
IL-1beta	0.81	0.18	0.31	0.60	0.007	0.17	0.13	0.002	0.92	1.41	0.194	0.826	0.869	0.827	26.96	25.44	<0.001	17.68	6.19	<0.001	11.68	15.55	0.362	0.374	0.009	0.106	<0.001	<0.001	0.130
MIP-1alpha	1.82	0.54	0.59	1.00	0.007	0.29	0.34	0.002	1.97	2.53	0.201	0.819	0.137	0.165	58.81	37.73	<0.001	40.63	14.24	<0.001	23.60	26.74	0.362	0.207	0.004	0.081	<0.001	<0.001	0.287
CTACK	383.74	107.84	704.29	268.40	0.017	537.18	356.67	0.775	361.24	97.06	0.831	0.143	0.016	0.705	10967.08	6259.12	<0.001	10834.17	3393.51	<0.001	3484.85	3969.94	0.482	0.456	<0.001	0.001	<0.001	<0.001	0.433
SDF-1alpha	83.01	34.43	138.75	47.12	0.033	122.86	34.71	0.099	190.43	37.99	0.010	0.432	0.102	0.058	2357.38	753.90	<0.001	2891.82	386.95	<0.001	908.63	1246.60	0.960	0.059	0.003	0.003	<0.001	<0.001	0.432
TNF-alpha	16.48	2.44	44.52	49.69	0.193	35.01	19.78	0.032	72.81	62.96	0.019	0.845	0.203	0.449	434.82	290.12	<0.001	325.33	163.85	<0.001	200.05	269.00	0.362	0.522	0.009	0.093	<0.001	<0.001	0.598
IL-13	2.94	0.57	21.76	9.85	0.001	21.27	8.04	0.004	22.10	6.58	0.011	0.922	0.777	0.990	75.13	60.61	<0.001	47.50	17.98	<0.001	34.46	36.68	0.131	0.241	0.017	0.107	<0.001	<0.001	0.793
IL-2	3.85	1.73	16.13	12.86	0.001	11.59	2.31	0.003	16.93	10.81	0.011	0.845	0.524	0.850	94.34	77.15	0.002	54.12	48.13	0.007	50.64	74.16	0.363	0.095	0.031	0.373	<0.001	0.051	0.990
beta-NGF	2.00	0.33	3.47	1.24	0.001	3.16	0.65	0.005	3.48	0.67	0.010	0.731	0.476	0.448	47.61	30.17	<0.001	53.22	13.96	<0.001	14.49	26.94	0.208	0.384	<0.001	0.002	<0.001	<0.001	0.695
basic-FGF	14.24	6.07	42.03	15.47	0.002	44.80	6.35	0.003	53.23	22.44	0.011	0.380	0.479	0.925	229.08	103.46	<0.001	154.90	71.89	<0.001	108.96	134.51	0.268	0.036	0.005	0.100	<0.001	<0.001	0.896
IFN-gamma	66.70	4.69	277.00	109.63	0.001	327.71	96.23	0.003	402.00	236.77	0.010	0.205	0.257	0.705	1045.33	595.16	<0.001	828.95	333.26	<0.001	519.18	548.59	0.035	0.570	0.016	0.123	<0.001	<0.001	0.695
G-CSF	27.60	4.94	34.67	17.32	0.448	30.99	15.98	0.775	54.13	27.86	0.136	0.696	0.322	0.257	406.91	172.93	<0.001	371.00	186.39	<0.001	170.85	199.04	0.190	0.346	0.002	0.020	<0.001	<0.001	0.512
IL-4	2.86	0.54	6.47	2.05	0.002	7.25	2.02	0.003	9.01	4.78	0.011	0.407	0.524	0.925	35.95	17.48	<0.001	28.06	10.15	<0.001	18.10	20.49	0.365	0.207	0.012	0.139	<0.001	<0.001	0.990
IL-7	4.52	0.91	6.14	4.50	0.515	9.57	5.31	0.032	10.20	8.83	0.087	0.143	0.396	0.507	56.70	42.63	<0.001	43.13	19.04	<0.001	32.28	39.19	0.362	0.499	0.046	0.107	<0.001	<0.001	0.598
MIF	554.46	115.89	2746.14	2300.92	0.030	1088.45	805.83	0.086	1203.04	1088.92	0.990	0.205	0.120	0.705	6704.86	8785.16	<0.001	3968.82	2085.60	<0.001	1519.66	1243.84	0.227	0.434	<0.001	0.006	0.041	0.002	0.601
IL-9	7.02	3.79	68.00	84.89	0.001	40.53	18.76	0.003	51.04	35.78	0.011	0.732	0.671	0.924	80.73	48.26	<0.001	55.12	37.70	<0.001	72.79	33.11	0.007	0.098	0.070	0.818	0.070	0.556	0.990
MIP-1 beta	20.22	7.20	85.34	26.86	0.001	75.68	14.11	0.003	79.21	20.58	0.011	0.696	0.671	0.990	191.45	148.52	<0.001	88.35	28.74	<0.001	176.87	115.12	<0.001	0.007	0.859	0.017	0.007	0.497	0.037
SCF	54.46	10.21	110.14	27.31	0.002	61.29	24.99	0.943	80.54	34.20	0.201	0.005	0.203	0.450	470.99	433.46	0.008	274.65	120.33	<0.001	162.74	112.44	0.035	0.374	0.026	0.045	0.004	<0.001	0.191
IL-1ra	111.83	19.39	192.57	100.31	0.065	182.57	48.78	0.015	289.50	210.24	0.033	0.732	0.571	0.705	808.00	508.89	<0.001	404.85	206.62	<0.001	289.18	358.30	0.688	0.007	<0.001	0.071	<0.001	0.002	0.695
IL-18	69.89	33.36	202.13	149.90	0.030	247.41	118.95	0.004	81.42	26.57	0.286	0.435	0.090	0.014	500.35	421.82	0.002	627.91	1222.49	0.007	120.18	43.30	0.044	0.256	<0.001	0.003	0.023	0.556	0.117
PDGF-bb	532.04	97.67	6109.80	2903.48	0.001	8835.57	2931.22	0.003	3551.25	700.71	0.011	0.118	0.090	0.008	3314.97	2131.40	0.002	1635.80	949.34	0.005	3445.35	4524.77	0.035	0.016	0.214	0.718	0.016	<0.001	0.151
IL-12p70	7.98	2.35	52.73	33.08	0.002	61.63	27.09	0.032	47.39	17.75	0.011	0.495	0.777	0.257	46.30	48.89	0.004	21.49	16.45	0.016	22.93	28.51	0.056	0.075	0.286	0.743	0.345	0.016	0.151
RANTES	798.53	160.94	7547.70	5505.60	0.001	8854.71	4907.89	0.003	5176.50	6619.39	0.011	0.558	0.120	0.131	4618.49	2745.17	<0.001	2947.19	1253.42	<0.001	6027.62	4280.65	<0.001	0.055	0.394	0.020	0.151	0.010	0.151
SCGF-beta	45616.56	18076.42	73158.57	33202.14	0.083	67878.64	45821.33	0.568	85503.11	94715.44	0.670	0.696	0.322	0.705	111894.60	51239.37	0.001	80444.73	23216.23	0.016	90322.47	25575.08	0.005	0.062	0.214	0.250	0.019	0.497	0.151

a: Compare between Control and H1N1 Day0-7 groups
b: Compare between Control and H1N1 Day8-14 groups
c: Compare between Control and H1N1 Day15- groups
d: Compare between H1N1 Day0-7 and H1N1 Day8-14 groups
e: Compare between H1N1 Day0-7 and H1N1 Day15- groups
f: Compare between H1N1 Day8-14 and H1N1 Day15- groups
g: Compare between Control and H7N9 Day0-7 groups
h: Compare between Control and H7N9 Day8-14 groups
i: Compare between Control and H7N9 Day15- groups
j: Compare between H7N9 Day0-7 and H7N9 Day8-14 groups
k: Compare between H7N9 Day0-7 and H7N9 Day15- groups
l: Compare between H7N9 Day8-14 and H7N9 Day15- groups
m: Compare between H1N1 Day0-7 and H7N9 Day0-7 groups
n: Compare between H1N1 Day8-14 and H7N9 Day8-14 groups
O: Compare between H1N1 Day15- and H7N9 Day15- groups

Supplementary Figure 2

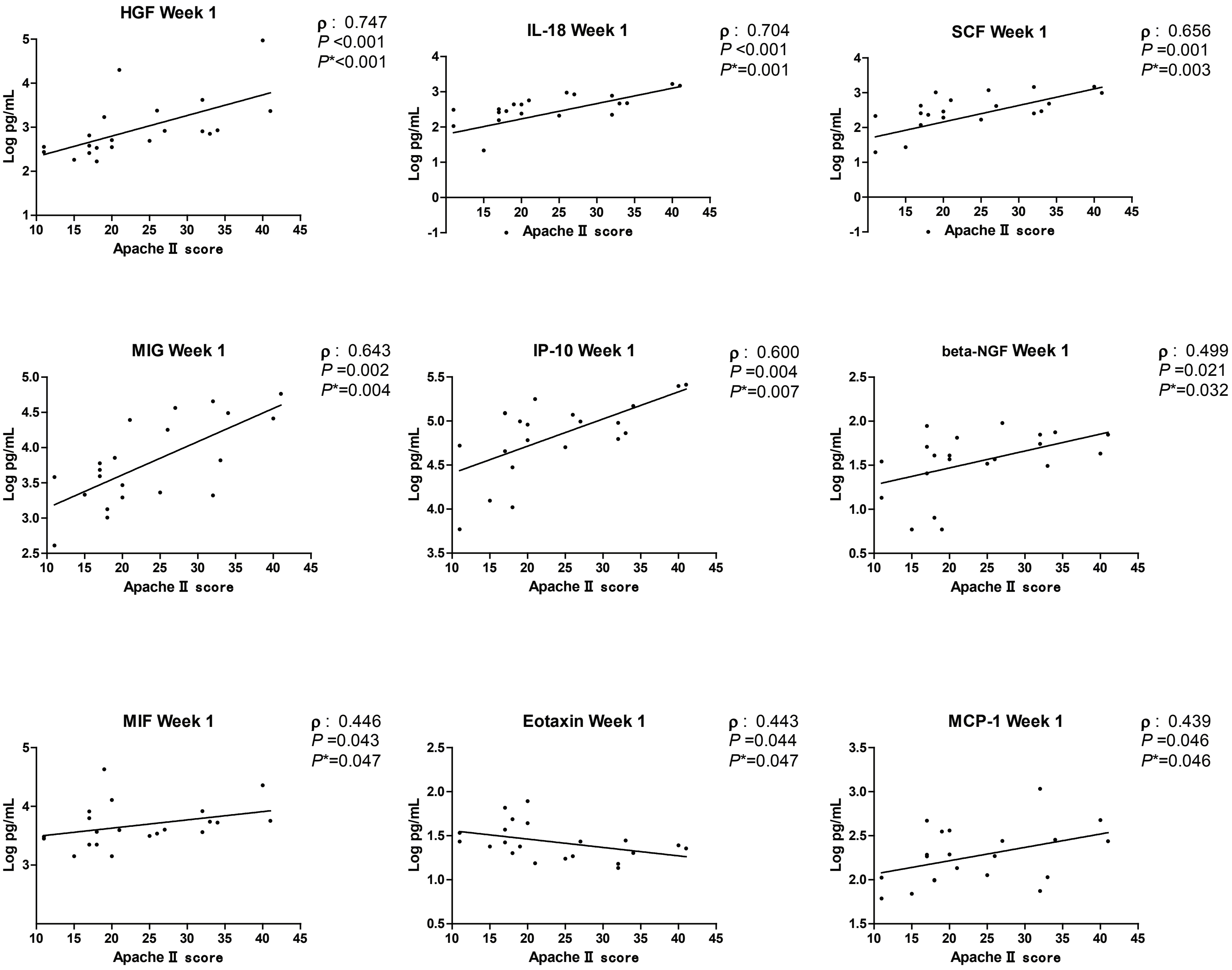

Supplementary Figure 3

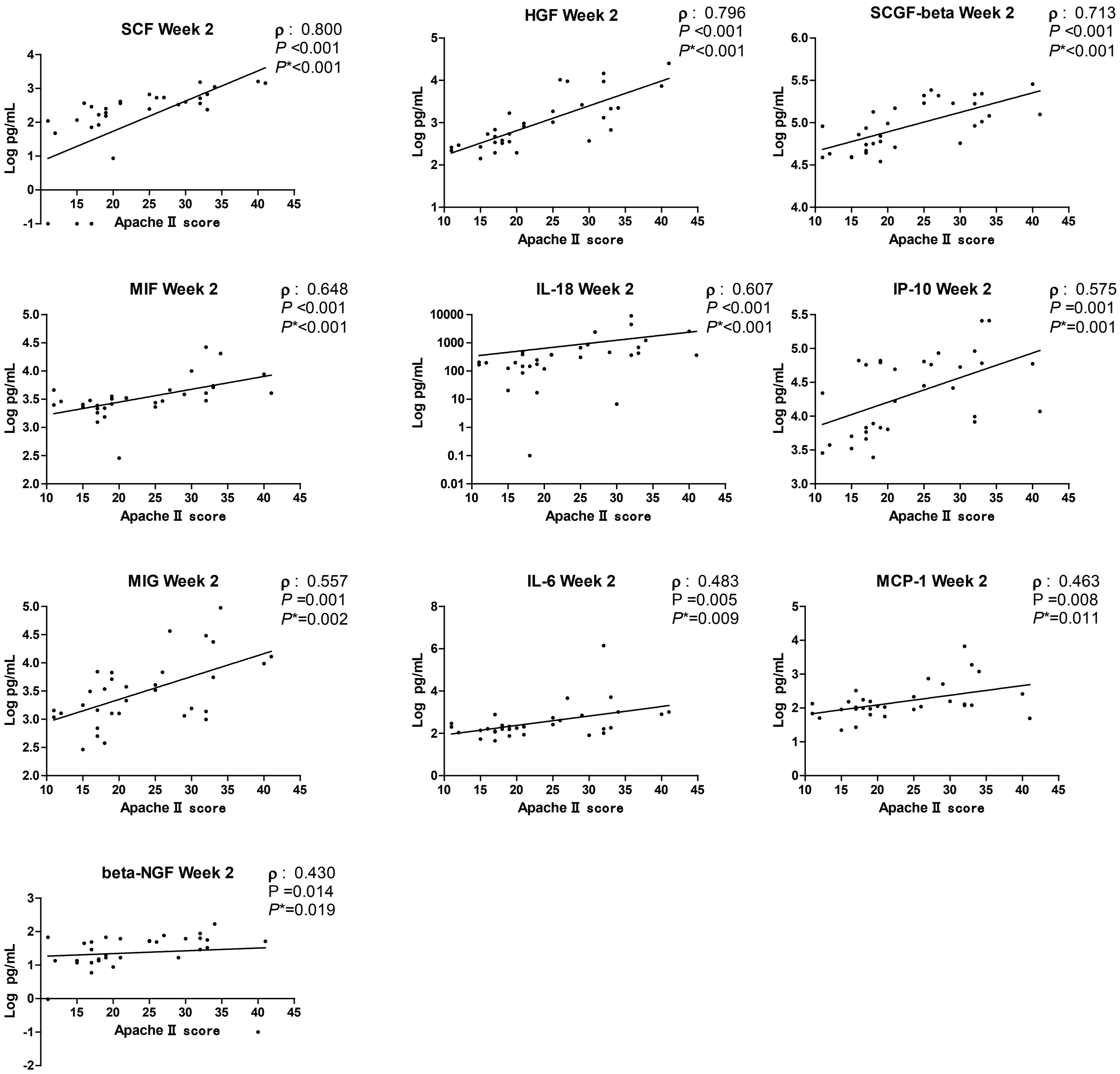

Supplementary Table 2. Outcomes in H7N9-infected Patients

Cyto/Chemokines	Week1					Week2				
	Hospitalized<28 days(n=12)		Death(n=5)		P Value	Hospitalized<28 days(n=16)		Death(n=8)		P Value
	Mean	SD	Mean	SD		Mean	SD	Mean	SD	
MIF	6788.97	11052.77	8350.28	7592.12	>0.05	2478.12	976.28	10230.90	8136.02	<0.001
MCP-1	185.02	119.67	395.86	373.37	>0.05	104.44	68.94	1143.97	2113.30	0.001
SCF	334.60	296.80	748.49	608.28	>0.05	144.21	129.21	762.14	530.10	0.001
HGF	2142.40	5417.31	19960.04	36874.91	>0.05	516.96	384.82	4824.59	4796.72	0.002
SCGF-beta	106784.14	57776.73	127463.78	47259.38	>0.05	73015.19	36486.71	151362.42	69428.71	0.004
IP-10	81233.46	58401.52	120058.92	73759.23	>0.05	19832.99	21839.53	70899.03	75499.28	0.008
IL-18	335.58	267.76	653.74	553.92	>0.05	193.28	142.20	2326.10	2917.72	0.008

Supplementary Table 3. ROC Curve of Cytokine/Chemokines in The Second Week H7N9-infected Illness Onset

Cyto/Chemokines	Area under the ROC curve	P value	Std. Errora	95% Confidence Interval	
				Lower Bound	Upper Bound
MIF	0.953	<0.001	0.041	0.874	1.000
SCF	0.93	0.001	0.050	0.831	1.000
MCP-1	0.906	0.001	0.060	0.788	1.000
HGF	0.891	0.002	0.073	0.747	1.000
SCGF-beta	0.867	0.004	0.075	0.721	1.000
IL-18	0.836	0.008	0.112	0.617	1.000
IP-10	0.836	0.008	0.082	0.676	0.996
IFN-gamma	0.777	0.03	0.111	0.561	0.994
beta-NGF	0.719	>0.05	0.123	0.477	0.960
IL-6	0.719	>0.05	0.121	0.482	0.955
MIG	0.664	>0.05	0.126	0.418	0.910
IL-8	0.637	>0.05	0.130	0.382	0.891
MIP-1beta	0.625	>0.05	0.138	0.355	0.895
SDF-1alpha	0.586	>0.05	0.131	0.329	0.843
CTACK	0.57	>0.05	0.138	0.299	0.842
MIP-1alpha	0.551	>0.05	0.138	0.281	0.820
IL-1ra	0.535	>0.05	0.135	0.271	0.799
IL-9	0.523	>0.05	0.133	0.262	0.785
IL-10	0.52	>0.05	0.139	0.247	0.792
G-CSF	0.504	>0.05	0.137	0.235	0.772
IL-7	0.477	>0.05	0.136	0.210	0.744
IL-5	0.453	>0.05	0.135	0.189	0.717
GM-CSF	0.422	>0.05	0.137	0.153	0.691
IL-1beta	0.422	>0.05	0.136	0.156	0.688
IL-4	0.414	>0.05	0.136	0.148	0.680
VEGF	0.406	>0.05	0.138	0.135	0.677
TNF-alpha	0.402	>0.05	0.136	0.135	0.670
IL-13	0.391	>0.05	0.128	0.141	0.641
IL-2	0.391	>0.05	0.129	0.138	0.644
basic-FGF	0.344	>0.05	0.126	0.097	0.590
IL-12p70	0.34	>0.05	0.127	0.091	0.589
PDGF-bb	0.336	>0.05	0.118	0.104	0.568
IL-17A	0.258	>0.05	0.104	0.054	0.461
RANTES	0.188	>0.05	0.093	0.006	0.369

Supplementary Figure 4

	IFN-gamma	MCP-1	IL-18	IP-10	HGF	MIF	SCF	SCGF-beta
IFN-gamma	1.00							
MCP-1		1.00						
IL-18			1.00					
IP-10	0.72	0.53	0.57	1.00				
HGF	0.45	0.48	0.67	0.68	1.00			
MIF	0.60	0.51	0.46	0.68	0.64	1.00		
SCF	0.49	0.51	0.62	0.75	0.77	0.70	1.00	
SCGF-beta		0.65	0.62	0.55	0.69	0.51	0.64	1.00

Supplementary Table 4. Comparison Among H7N9, 2009 S-OIV, URTI, H5N1 and SARS Induced Plasma Chemokine and Cytokine Levels.

	H7N9 (n=24)	P[1]	H1N1 (n=14)	P[2]	URTI(n=15) [Ref18,27]	H5N1(n=16) [3] [Ref12]
**MCP-1**	2.3(1.8-3.0)	< 0.001	1.5 (-0.7-2.9)	< 0.001	1.6 (und-2.1)	2.4 (1.5–4.0)
**IP-10**	5.0(3.8-5.4)	< 0.001	1.7 (0.4-4.6)	< 0.001	3.5 (2.3-4.2)	5.1 (3.5–6.3)
**IFN-γ**	3.0(2.6-3.4)	< 0.001	2.0 (0.4-3.0)	< 0.001	2.0 (und-2.4)	2.0 (und.–4.2)
**IL-6**	2.3(1.7-3.6)	< 0.001	1.5 (0.6-1.9)	< 0.001	1.2 (und-1.6)	2.1 (und.–3.7)
**MIG**	3.7(2.6-4.8)					4.3 (3.1–5.2)
**IL-8**	2.4(2.0-2.8)	< 0.001	1.1 (0.2-2.4)	< 0.001	0.7 (und-1.5)	2.0 (0.7–3.2)
**IL-10**	2.1(1.1-2.5)	< 0.001	0.2 (0.2-2.0)	< 0.001	und (und-1.4)	1.5 (und.–2.8)

Levels are given as median log pg/mL (range). URTI., upper respiratory tract infection. und., undetectable.

[1]Comparison between H7N9 (0-7 d of illness onset) and 2009 swine flu (0-8 d of illness onset) patients. [2]Comparison between H7N9 (0-7 d of illness) and 2009 URTI patients. [3] The blood specimens of patients infected with H5N1 were obtained 4-8 d of illness onset.

	H7N9		SARS [Ref28]	
	**0-7 d of illness onset (n=24)**	**8-14 d of illness onset (n=11)**	**0-7 d of illness onset (n=126)**	**8-14 d of illness onset (n=129)**
**MCP-1**	185.1(184.1)	185.1(184.1)	48.8 (26.0–94.0)	31.6 (14.9–68.0)
**IP-10**	93096.4(83859.1)	93096.0(83862.0)	4020 (2520–6265)	2695 (945–4760)
**RANTES**	4361.8(3880.8)	4361.8(3880.8)	29 800 (18 900–33 720)	32 500 (16 200–36 600)
**MIG**	5418.1(20870.9)	5418.1(20871.2)	540 (375–918)	612 (360–1032)
**IL-8**	265.0(161.2)	265.0(161.2)	11.7 (6.6–31.7)	8.9 (5.4–32.8)

Levels are given as median pg/mL (IQR).

	H7N9(0-14 d of illness onset, n=35)	SARS(progressive stage, n=23) [Ref17]
**MCP-1**	194.2±189.1	475±1000
**IP-10**	83245.0±61267.3	8100±2300
**IFN-γ**	977.3±544.4	85±35
**IL-6**	350.0±726.9	120±160
**IL-8**	270.0±123.1	750±1420
**MIP-1α**	53.1±33.8	1790±2880

Levels are given as means pg/mL ± SD.

Figure Legend

Supplementary Figure 1.Hypercytokinemia in Avian Influenza A (H7N9) Virus-Infected Patients
A total of 46 patients infected with the avian influenza A (H7N9) virus, 21 patients infected with the swine-origin influenza A (H1N1) virusand 6 healthy controls were recruited for this study. Their plasma levels of chemokines and cytokines at 0-7 days (n=24), 8-14 days (n=11) and 15 days (n=11) after the onset of the disease were measured. Detailed information is shown in supplementary table 1. $^{*}P < 0.05$,$^{**}P < 0.01$, $^{***}P < 0.001$.

Supplementary Figure 2.The APACHE II Score was Correlated with the Plasma Levels of Chemokines and Cytokines and with Clinical Characteristics in The First Week of Disease Onset in Avian Influenza A (H7N9) Virus-Infected Patients.
Correlations between the APACHE II score and the plasma chemokine and cytokine (HGF, IL-18, SCF, MIG, IP-10, IL-6, MIF, Eotaxin, MCP-1) levels in the first week of influenza H7N9 infection for 21 patients.Spearman's rank correlation analysis (ρ), *P* value and the Benjamini& Hochberg multiple testing correction (*P**) to control for the false discovery rate (FDR) areshown in each graph.

Supplementary Figure 3.The APACHE II Score was Correlated with the Plasma Levels of Chemokines and Cytokines and with Clinical Characteristics in the Second Week of Disease Onset in Avian Influenza A (H7N9) Virus-Infected Patients.
Correlations between the APACHE II score and the plasma chemokine and cytokine levels (SCF, HGF, SCGF-β, MIF, IL-18, IP-10, MIG, IL-6, MCP-1, β-NGF) in the second week of influenza H7N9 infection in 32 patients.Spearman's rank correlation analysis (ρ), *P* value and the Benjamini& Hochberg multiple testing correction (*P**) to control for the false discovery rate (FDR) areshown in each graph.

Supplementary Figure 4.Correlation Heatmap of Plasma Cytokine/Chemokines Levels in the Second Week of H7N9-infected Illness Onsets.
Spearman's rank correlation analysis between the plasma chemokine and cytokine levels (SCF, HGF, SCGF-β, MIF, IL-18, IP-10, IFN-γ, MCP-1) in the second week of influenza H7N9 infection. The blank represents the Spearman's rank correlation analysis' P value > 0.05.

Supplementary Table 1. Cytokine/chemokine Comparison Among 6 Healthy Controls, 21 H1N1-infected Patients and 46 H7N9-infected Patients.
Supplementary Table 2. Outcomes in H7N9-infected Patients
Supplementary Table 3. ROC Curve of Cytokine/Chemokinesduringthe Second Week H7N9 Infection
Supplementary Table 4. Comparison among H7N9, 2009 S-OIV, URTI, H5N1 and SARS Induced Plasma Chemokine and Cytokine Levels
